# Model selection criteria for dynamic brain PET studies

**DOI:** 10.1186/s40658-017-0197-0

**Published:** 2017-12-06

**Authors:** Sandeep S. V. Golla, Sofie M. Adriaanse, Maqsood Yaqub, Albert D. Windhorst, Adriaan A. Lammertsma, Bart N. M. van Berckel, Ronald Boellaard

**Affiliations:** 1 Department of Radiology and Nuclear Medicine, VU University Medical Center, P.O. 7057, 1007 MB Amsterdam, The Netherlands; 2 Department of Nuclear Medicine and Molecular Imaging, University of Groningen, University Medical Center Groningen, Groningen, The Netherlands

**Keywords:** Positron emission tomography, Brain imaging, Molecular imaging, Pharmacokinetics, Imaging

## Abstract

**Background:**

Several criteria exist to identify the optimal model for quantification of tracer kinetics. The purpose of this study was to evaluate the correspondence in kinetic model preference identification for brain PET studies among five model selection criteria: Akaike Information Criterion (AIC), AIC unbiased (AIC_C_), model selection criterion (MSC), Schwartz Criterion (SC), and F-test.

**Materials and Methods:**

Six tracers were evaluated: [^11^C]FMZ, [^11^C]GMOM, [^11^C]PK11195, [^11^C]Raclopride, [^18^F]FDG, and [^11^C]PHT, including data from five subjects per tracer. Time activity curves (TACs) were analysed using six plasma input models: reversible single-tissue model (1T2k), irreversible two-tissue model (2T3k), and reversible two-tissue model (2T4k), all with and without blood volume fraction parameter (*V*_B_). For each tracer and criterion, the percentage of TACs preferring a certain model was calculated.

**Results:**

For all radiotracers, strong agreement was seen across the model selection criteria. The F-test was considered as the reference, as it is a frequently used hypothesis test. The F-test confirmed the AIC preferred model in 87% of all cases. The strongest (but minimal) disagreement across regional TACs was found when comparing AIC with AIC_C_. Despite these regional discrepancies, same preferred kinetic model was obtained using all criteria, with an exception of one FMZ subject.

**Conclusion:**

In conclusion, all five model selection criteria resulted in similar conclusions with only minor differences that did not affect overall model selection.

**Electronic supplementary material:**

The online version of this article (10.1186/s40658-017-0197-0) contains supplementary material, which is available to authorized users.

## Background

The full potential of a novel PET tracer can only be achieved when the uptake or clearance of the tracer can be extracted from the normal activity distribution of the dynamic PET data. To evaluate this, a pharmacokinetic model describing the tracer in vivo kinetics is necessary. With a kinetic model, the in vivo physiological behaviour of the tracer both in tissue and blood can be described. The model that best describes the kinetic behaviour of the tracer may be used to reproducibly and reliably quantify tracer distribution or binding. Finding the optimal model can, however, be a challenge.

Several criteria exist to identify the preferred kinetic model that provides the best fit to the time course of the tissue tracer concentration [1]^,^ [2]^,^ [3]^,^ [4]^,^ [5]. Each of these criteria ranks the quality of the model fit and thus provides a means for model selection. These criteria are all based on the goodness of fit (sum of squared differences between measured and estimated data, also termed residuals) and the complexity of the model (number of fit parameters). In addition, both number of frames and weighting factors applied to residuals are taken into account. The various criteria have substantial similarities in their equations and parameters used (Eqs. 1–4). For example, Eq. 3 can be written as the difference between ln of nominator and denominator; this now is quite similar to other equations. The only difference is in the penalty parameter (added based on the complexity of model) in these equations. We therefore hypothesize that these criteria would result in (overall) comparable model preferences in actual clinical data. To the best our knowledge, a direct comparison of their performance in clinical PET brain studies has never been reported and the selection of the criterion used is frequently a matter of debate.

The purpose of the present study was to evaluate the similarity and/or difference in model preference for several commonly used criteria: Akaike Information Criterion (AIC) [6], [1], AIC unbiased (AIC_C_) [7], [2], Model Selection Criterion (MSC) [8], [3], Schwartz Criterion (SC) [9], [4], and finally validate them against the F-test [10]^,^ [5]. To cover a range of kinetics, these criteria were used to identify the preferred model for six tracers with known differences in kinetic (in vivo) properties.

## Methods

Six neuroPET tracers with different kinetic properties were evaluated: [11C]Flumazenil ([11C]FMZ), [11C]N-(2-chloro-5-thiomethylphenyl)-N′-(3-methoxy-phenyl)-N′-methylguanidine ([^11^C]GMOM), [^11^C](1-[2-chlorophenyl]-N-methylN-[1-methyl-propyl]-3-isoquinoline carboxamide) ([^11^C]PK11195), [^11^C]Raclopride, [^18^F]Fluorodeoxyglucose ([^18^F]FDG), and [^11^C]Phenytoin ([^11^C]PHT). GABA (A) receptor binding, NMDA receptor binding, TSPO binding, dopamine receptor binding, metabolic rate of glucose, and function of Pgp transporters can be deduced using the following PET tracers: [^11^C]FMZ, [^11^C]GMOM, [^11^C]PK11195, [^11^C]raclopride, [^18^F]FDG, and [^11^C]PHT, respectively. Additional file 1: Figure S1 illustrates typical whole brain gray matter time activity curves (TACs) of the six tracers. A full description of the original studies has been reported previously for [^11^C]FMZ [11], [^11^C]PK11195 [12], [^11^C]Raclopride [13], [^18^F]FDG [14], [^11^C]GMOM [15], and [^11^C]PHT [1]. For each tracer, PET scan data from five subjects were included. Subjects were randomly selected from existing databases, and researchers had no information on group status (both healthy subjects and patients). Five subjects per tracer reflect the number of subjects often included in first-time studies examining tracer kinetics. Each of these subjects had a T1-weighted magnetic resonance imaging (MRI) scan and a metabolite corrected arterial input function was also available. For scanner properties, attenuation correction, scan duration, and reconstruction methods, see Table 1. All scans were corrected for dead time, randoms, scatter, and decay. The original studies had all been approved by the Medical Ethics Review Committee of the VU University Medical Center. All subjects had provided written informed consent after complete explanation of the study procedures.

**Table 1:** Overview of retrospective datasets used

	[^11^C]FMZ	[^11^C]PK11195	[^11^C]Raclopride	[^18^F]FDG	[^11^C]GMOM	[^11^C]PHT
Scanner	ECAT EXACT HR+	ECAT EXACT HR+	ECAT EXACT HR+	ECAT EXACT HR+	Gemini TF-64 PET/CT	Gemini TF-64 PET/CT
Attenuation correction	10 min two-dimensional transmission scan	10 min two-dimensional transmission scan	10 min two-dimensional transmission scan	10 min two-dimensional transmission scan	Low-dose CT	Low-dose CT
Scan duration (min)	60	60.5	60	60	90	60
Number of frames	16	22	20	39	22	19
Reconstruction method	OSEM	FORE+ 2D-filtered back projection algorithm	FORE+ 2D-filtered back projection algorithm	OSEM	3-D RAMLA	3-D RAMLA
MRI	Siemens Sonata 1.5 T	Siemens 1.0 T IMPACT	Siemens Sonata 1.5 T	Siemens Magnetom Vision	Siemens Sonata 1.5 T	Siemens Sonata 1.5 T

ECAT EXACT HR+ scanner (CTI/Siemens, Knoxville, TN, USA). Gemini TF-64 PET/CT scanner (Philips Medical Systems, Cleveland, OH, USA). Philips Intera 1.5 T scanner (Philips Medical Systems, Best, The Netherlands). Siemens Sonata 1.5 T & Siemens 1.0 T IMPACT (Siemens, Erlangen, Germany)

*3-D RAMLA* three-dimensional row action maximum likelihood reconstruction algorithm, *OSEM* ordered subset expectation maximization

For anatomical delineation of volumes of interest (VOIs), MRI was used. Subjects’ T1-weighted MRI scan was co-registered with the summed PET data using VINCI software (Cologne, Germany). The co-registered MRI scan was segmented into gray matter, white matter, and extra-cerebral fluid, and TACs were extracted using PVElab [16] in combination with the Hammers template [17]. Sixty-eight gray matter VOIs were delineated onto MRI, i.e. 68 TACs were extracted per subject. TACs were analysed using six plasma input models: a reversible single-tissue model (1T2k), an irreversible two-tissue model (2T3k), and a reversible two-tissue model (2T4k), all three both with and without additional blood volume parameter (*V*_B_) [18].

For all six tracers, the preferred models across TACs were obtained using various model selection criteria. Equations below show the implementation of the various model selection criteria. Akaike Information Criterion (AIC) [[6]; Equation (Eq.) 1], AIC unbiased (AIC_C_) [[7]; Eq. 2], Model Selection Criterion (MSC) [[8]; Eq. 3], Schwartz Criterion (SC) [[9]; Eq. 4], and the F-test [[10]; Eq. 5].

1 $$ \mathrm{AIC}=n\ \ln \left({\sum}_i{w}_i{\left(y\left({t}_i\right)-\widehat{y}\left({t}_i\right)\right)}^2/n\right)+2p $$

2 $$ {\mathrm{AIC}}_{\mathrm{C}}=n\ \ln \left({\sum}_i{w}_i{\left(y\left({t}_i\right)-\widehat{y}\left({t}_i\right)\right)}^2/n\right)+2p+\frac{2p\left(p+1\right)}{n-p-1} $$

3 $$ \mathrm{MSC}=\ln \frac{\sum_i{w}_i{\left[y\left({t}_i\right)-\overline{y}\left({t}_i\right)\right]}^2}{\sum_i{w}_i{\left[y\left({t}_i\right)-\widehat{y}\left({t}_i\right)\right]}^2}-2p/n $$

4 $$ \mathrm{SC}=n\ \ln \left({\sum}_t{w}_i{\left[y\left({t}_i\right)-\widehat{y}\left({t}_i\right)\right]}^2/n\right)+p\ln (n) $$

5 $$ \mathrm{F}=\frac{\left({Q}_1-{Q}_2\right)/\left({p}_2-{p}_2\right)}{Q_{2/}\left(n-{p}_2\right)} $$

where *n* represents the number of observations, *p* the number of parameters, *w*_i_ the weight applied to residual of *i*th acquisition, *y* are the measured values (PET scan), *ŷ* are the predicted (fitted) values, $\bar{y}$ is the mean of the measured values, and *t*_i_ time of *i*th acquisition. For the F-test, *Q*_1_ represents the sum of squares for the model with *p*1 parameters, and *Q*_2_ the sum of squares of model with *p*2 parameters, where *p*1 < *p*2 is required. *w*_i_ (*w*_i_ = 1/*σ*_i_^2^) was estimated using the following equation [19]:

${\sigma}^2=\alpha \bullet \mathrm{dcf}\bullet \mathrm{dcf}\frac{T}{L\bullet L}$, where *σ* ^2^represents the variances for each frame and is calculated based on the whole scanner trues counts (*T*); dcf is the decay correction factor, *L* represents frame length, and *α* is the proportionality constant signifying the variance level.

For, AIC, AIC_C_, and SC, a lower value implies a better fit. Therefore, the lowest value obtained from the model selection criterion indicates the preferred kinetic model, i.e. the model that provides the best fit to a TAC with the smallest number of model parameters. For MSC, a higher value implies a better fit and hence the highest value will be indicative of the most appropriate model. F-test directly compares two models and returns a F-statistic with *p*2-*p*1 and *n*-*p*2 degrees of freedom. If this F-statistic is larger than the tabulated value at a specified *p* value, the complex model has a better fit (with a significance of *p* < 0.05).

Agreement of the model selection criteria was evaluated by calculating the percentage preference for a kinetic model across all TACs. This was performed for all subjects and for each model selection criteria and tracer. The F-test was considered as the reference for model comparison as it is a frequently used hypothesis test [20]^,^ [21]. This F-test, however, is difficult to apply when comparing multiple models, subjects, TACs, and tracer studies. For example, in the present study, this would have resulted in 61,200 comparisons (30 comparisons per tracer per subject per VOI). Therefore, a more pragmatic approach was followed by first identifying the most likely plausible model based on known tracer kinetics, visual inspection of the fits, and AIC results, which can be calculated very efficiently. Next, the F-test was applied to determine if this model was the preferred one (per TAC) when compared to all other models. Finally, regional agreement/disagreement of AIC_C_, SC, and MSC to AIC and eventually F-test was evaluated. To examine if any existent disagreement between model selection criteria was a general effect or driven by a single subject, the frequency of model preferences per subject were also evaluated for each model selection criteria. Finally, in order to examine the effect of VOI size (assuming that smaller VOIs result in TACs with higher noise levels) on the model selection criteria, similar analyses were performed for VOIs with sizes smaller and larger than 5 mL separately.

## Results

**Fig. 1:**
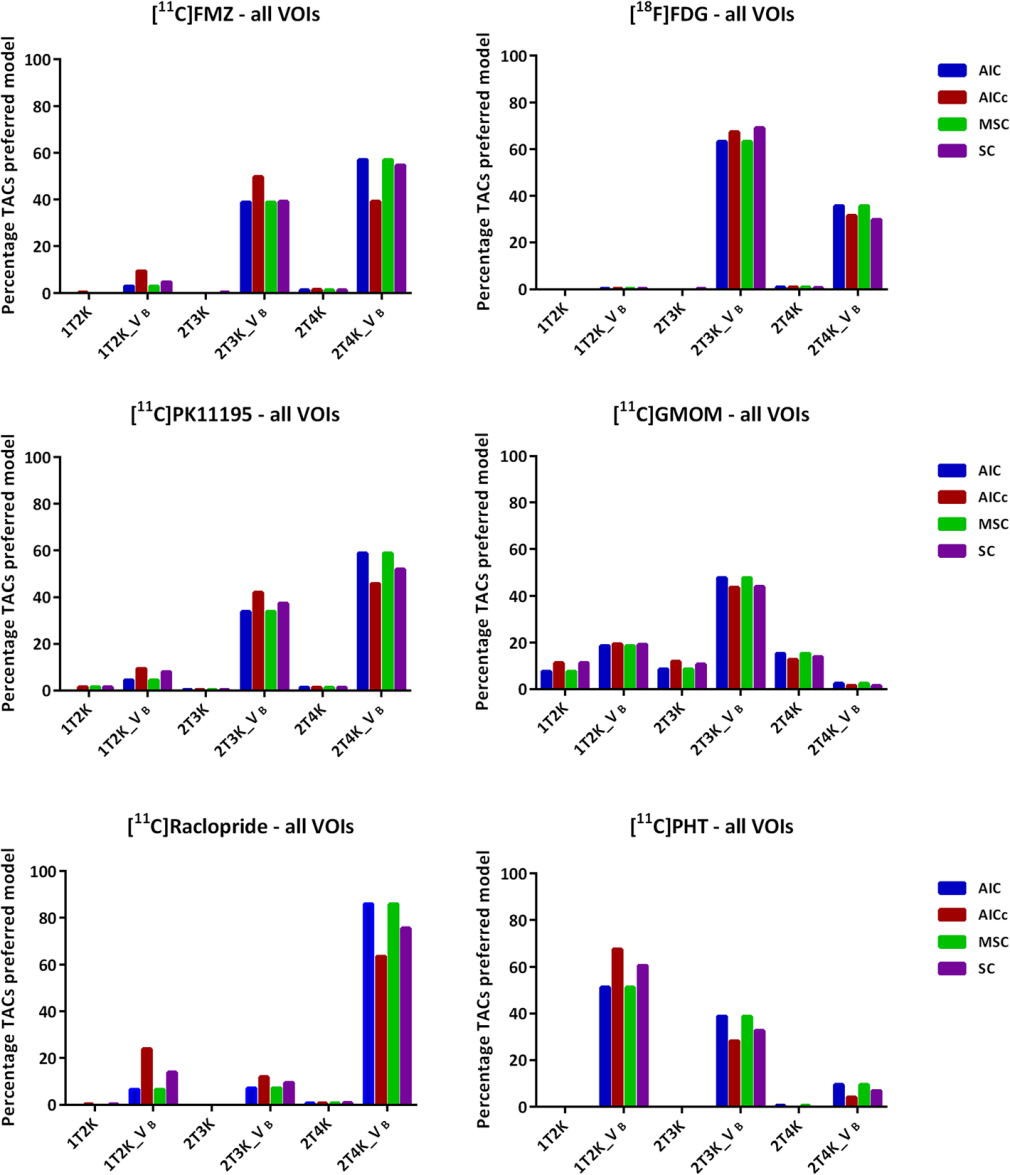
Model preference (percentage of all TACs) per selection criterion for the six brain PET radiotracers

For all six radiotracers, strong agreement was observed between the different model selection criteria when examining the frequency of model preferences across all TACs (Fig. 1). Only one small deviation was observed for FMZ: AIC_C_ preferred 2T3k_*V*_B_, whereas the other model selection criteria preferred 2T4k_*V*_B_. The reduced regional agreement for FMZ when comparing AIC with AIC_C_ turned out to be an 18% difference in model preference. When examining within subject preference, it was shown that this difference was mainly driven by one subject. For this subject, 2T3k_*V*_B_ was preferred according AIC_C_ and F-test where AIC, MSC, and SC all preferred 2T4k_*V*_B_ for this subject. For the other remaining four FMZ studies, all five criteria agreed on 2T4k_*V*_B_ being the preferred model for FMZ.

Even though all model selection criteria agreed on the preferred model across TACs (with exception of one FMZ study), slight disagreements between criteria did exist. The F-test confirmed the AIC preferred models within single subjects for 87% of all cases. AIC and MSC gave exactly the same model preferences for all the tracers studied. Regional disagreement of only 13 and 7% for AIC_C_ and SC, respectively, with AIC was observed for [^11^C]PK11195. In case of [^11^C]Raclopride, regional disagreement of 22% when comparing AIC with AIC_C_ and 10% between AIC and SC was observed. For [^18^F]FDG, relatively low disagreement of 4 and 6% for AIC_C_ and SC, respectively, was observed. A relatively low (4%) disagreement of AIC with both AIC_C_ and SC (4%) was obtained for [^11^C]GMOM. A regional disagreement of 16% for AIC_C_ and 9% for SC with AIC was seen for [^11^C]PHT. Despite regional differences, and between subject deviations, all model selection criteria showed strong agreement in model preference.

**Fig. 2:**
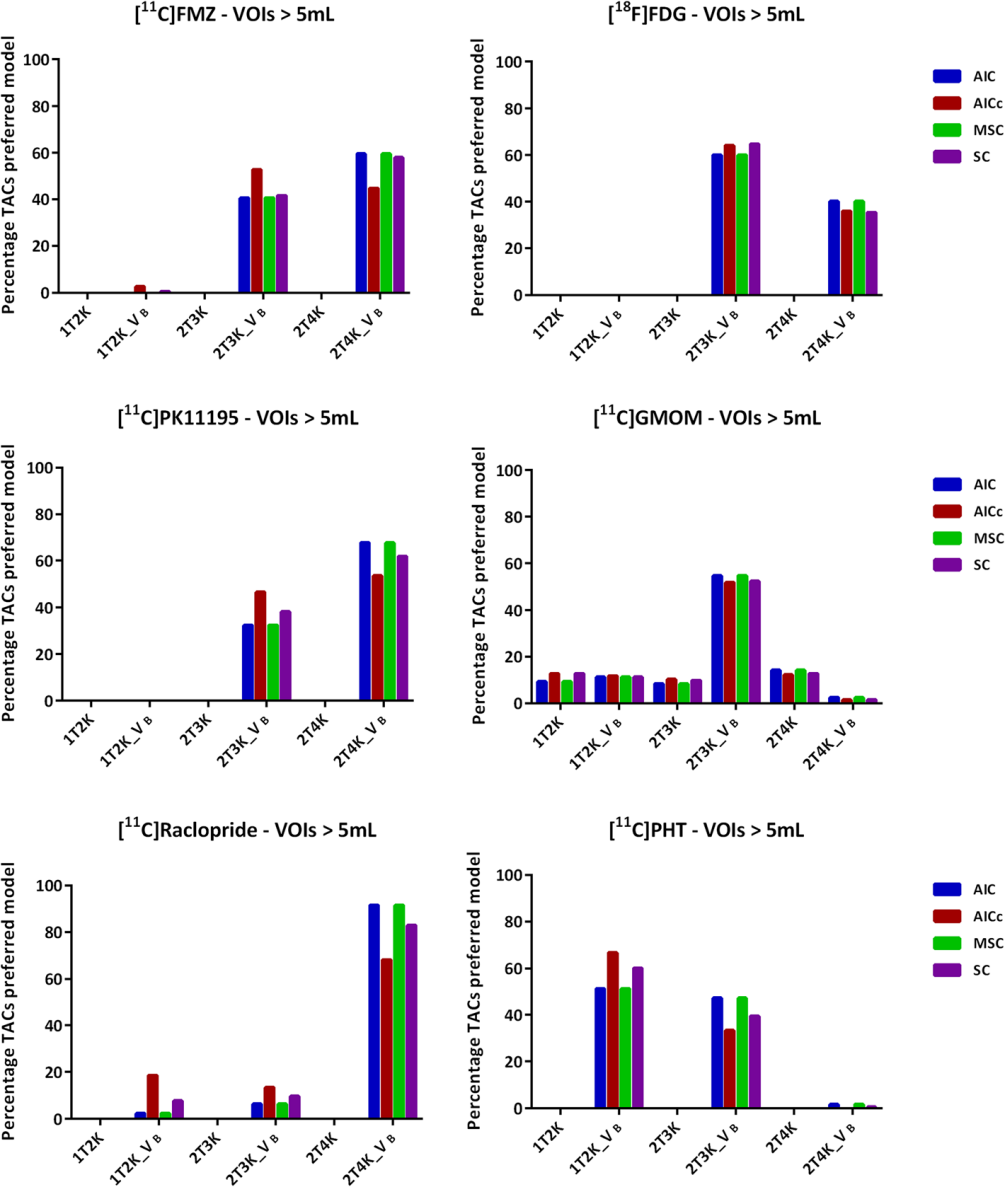
Model preference (percentage of all TACs ≥ 5 mL) per selection criterion for the six brain PET radiotracers

**Fig. 3:**
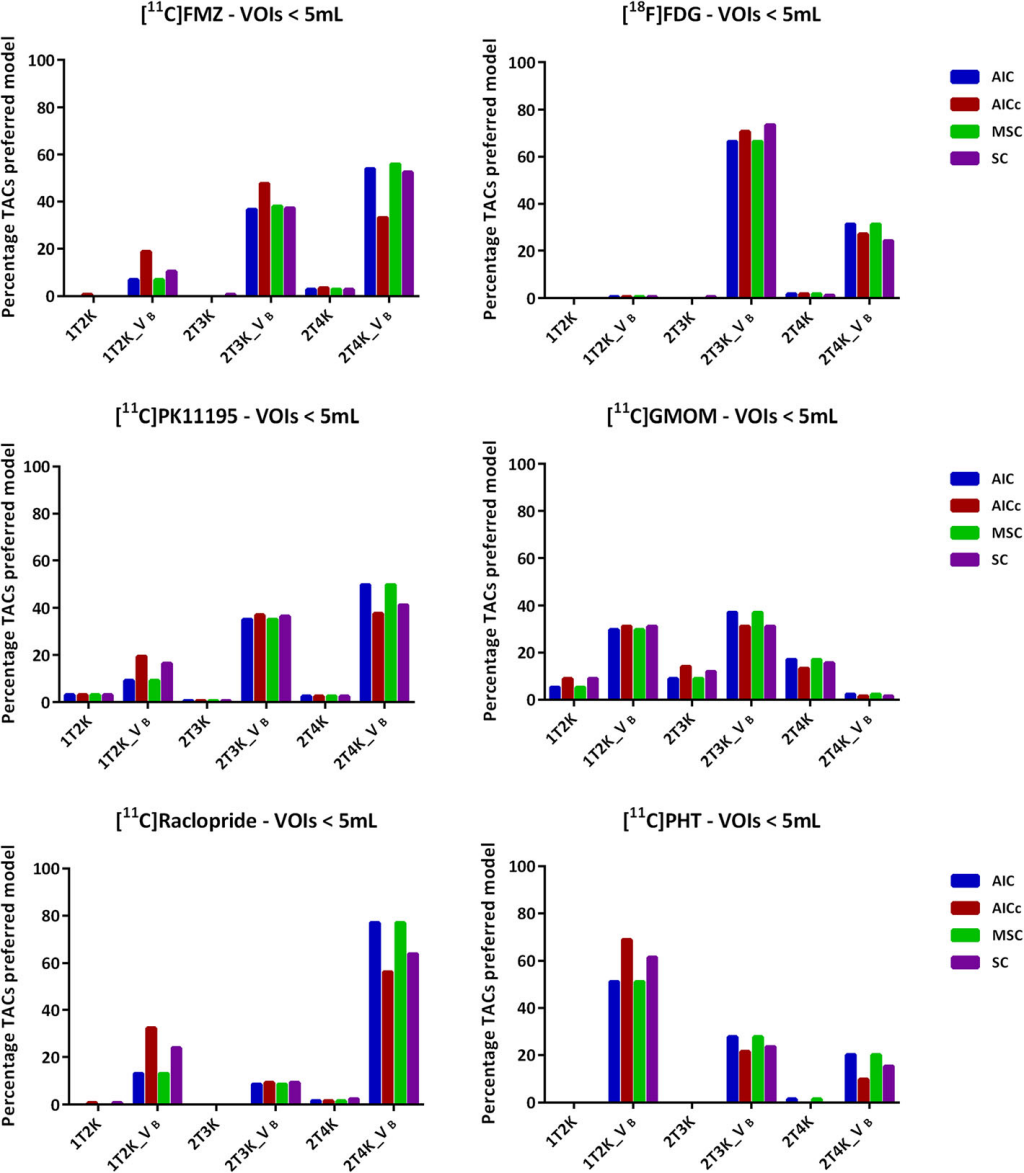
Model preference (percentage of all TACs < 5 mL) per selection criterion for the six brain PET radiotracers

Effect of noise on the model preference can be observed in Figs. 2 and 3. For all the tracers, a variation in the model preference is observed with varying VOI size, which is primarily due to noise. However, the VOI size (either smaller or larger than 5 mL), in general, did not have a notable effect on the agreement of the model selection criteria (Figs. 2 and 3).

## Discussion

The aim of the present study was to assess the overall agreement and/or disagreement in model preference of five commonly used model selection criteria. The model selection criteria evaluated are all based on similar assumptions, possibly explaining that the choice for a certain model selection criterion hardly affected identification of the preferred model. For a mathematical evaluation of the underlying assumptions of model selection criteria and their considerations, see [22], [23], [24].

In short, small differences between criteria exist with respect to the penalty applied for the number of parameters and observations (frames). AIC, AIC_C_, SC, and MSC all include an extra penalty term for the number of parameters, trying to account for the possibility of over-fitting the model. The penalty used in AIC_C_ seems to be stronger than that used in SC and MSC when compared to AIC. AIC becomes strongly biased when the ratio of model parameters to observations (acquisition frames) increases, for which AIC_C_ tries to correct. When the number of observations increases, AIC_C_ converges to AIC. Due to the stronger penalty, AIC_C_ seems to prefer the models with less parameters when the ratio is bigger. For [^11^C]FMZ and [^11^C]PHT, both with < 20 observations, AIC_C_ indeed preferred the model with less parameters slightly more frequently compared with the other criteria. For [^11^C]PHT, this was reflected in a regional disagreement between AIC and AIC_C_ of 16% of the TACs. For FMZ, this resulted in AIC_C_ identifying a different kinetic model when examining all VOIs across all subjects as illustrated in Fig. 1. However, this difference in model preference was mainly driven by a single subject where AIC_C_ disagreed strongly with the other model selection criteria, which was confirmed by the F-test. For the remaining four subjects, AIC_C_ and F-test preferred the same model for FMZ as did the other criteria.

For [^11^C]PK11195, [^11^C]raclopride, [^18^F]FDG, and [^11^C]FMZ, the F-test did not agree with AIC for only four individual (subject) cases (one subject from each tracer study). For [^11^C]PHT and [^11^C]GMOM, the F-test agreed with AIC in all cases. The overall agreement for all tracers and subjects was 87% between AIC and F-test. However, on a (tracer) study level, this did not affect the model preference for that tracer. The F-test is designed to directly compare two models and is especially suited for this purpose. AIC and MSC yielded identical results. Compared with AIC, MSC includes normalization and is therefore independent of scaling of the data points. The normalization incorporated in MSC, however, seems to have little effect for the data described in this study.

Even though the model selection criteria showed strong overlap, it is not always straightforward which model provides the best fit for the data. For example, ~ 30% of all TACs clearly identified 2T4k_*V*_B_ for [^18^F]FDG, whereas the kinetics of all other TACs were described by 2T3k_*V*_B_.

A total of 68 gray matter VOIs were included per subject, implying that the analysis comprised of both receptor-rich and receptor-devoid VOIs (which varies depending on the target of interest/tracer) and thus the observed regional differences in model preferences (Figs. 1, 2, and 3). In addition to the receptor density, another reason for regional differences in the same subject for the same tracer could be the size of VOI. Smaller VOI tends to have lower counts and thereby higher noise, which makes it difficult to define the optimal pharmacokinetic model. Figure 2 illustrates the impact of noise on the model selection when using different model selection criteria. Noise could be a probable cause for the observed differences between Figs. 2 and 3.

Preferred model can be affected by several factors such as in vivo kinetics of the VOI (underlying receptor density), subject status, input function (particularly parent fraction and metabolites), tracer free fraction, motion, and even the scan duration. It should be noted that a description of tracer kinetics and the identification of the preferred kinetic model using model selection criteria is only one of the many evaluations needed to identify the optimal model. For example, in order to determine the optimal kinetic model, several other datasets and studies are required such as test-retest data, data for both healthy controls and diseased subjects, data correlating model results with pathology, and/or outcome (disease duration, cognitive scores, or survival). Also, the intended application, e.g. differential diagnosis or response assessment, may or may not allow for the use of simplified models or methods. Moreover, formulating a hypothesis on the preferred kinetic model based on physiological properties of the tracer, e.g. derived from preclinical studies, is advised.

The number of subjects included for each tracer might be a limitation for this study; however, typically first in man PET studies are limited in subject number and often restricted to healthy subjects. In these circumstances, use of model selection criteria provides a first indication of one or more suitable candidate kinetic models which should be further developed and evaluated. Even though both healthy controls and patients were included for the analysis, no impact on the conclusions is expected, as over a wide range of kinetics with quite consistent results in the performance of the various criteria was observed. Therefore, possible changes in tracer kinetics in healthy versus diseased subjects would likely have little impact on the performance of these criteria, but should be verified in individual cases.

## Conclusions

All model selection criteria tested resulted in similar conclusions with only minor, non-relevant differences in overall observed model preference.

## Additional file

Additional file 1: Figure S1. Typical whole brain gray matter TACs of all the six brain PET radiotracers. (TIFF 43 kb)

## Abbreviations

[^11^C]PHT: [^11^C]Phenytoin
[^18^F]FDG: [^18^F]Fluorodeoxyglucose
1T2k: Reversible single-tissue model
2T3k: Irreversible two-tissue model
2T4k: Reversible two-tissue model
AIC: Akaike Information Criterion
dcf: Decay correction factor
FMZ: Flumazenil
GMOM: N-(2-Chloro-5-thiomethylphenyl)-N′-(3-methoxy-phenyl)-N′-methylguanidine
*L*: Frame length
MSC: Model selection criterion
*N*: Number of observations
*P*: Number of parameters
PK11195: (1-[2-Chlorophenyl]-N-methylN-[1-methyl-propyl]-3-isoquinoline carboxamide)
*Q*_1_: Sum of squares for the model with p1 parameters
*Q*_2_: Sum of squares of model with p2 parameters
SC: Schwartz Criterion
T: Whole scanner trues counts
TACs: Time activity curves
*t*_i_: Time of *i*th acquisition
*V*_B_: Blood volume parameter
VOI: Volumes of interest
*w*_i_: Weight applied to residual of *i*th acquisition
$\bar{y}$: Mean of the measured values
*y*: Measured values (PET scan)
*ŷ*: Predicted (fitted) values
*α*: Proportionality constant signifying the variance level
σ^2^: Variances for each frame
